# Impact of Socioeconomic Status on Presentation and Outcomes in Colorectal Peritoneal Metastases Following Cytoreduction and Chemoperfusion: Persistent Inequalities in Outcomes at a High-Volume Center

**DOI:** 10.1245/s10434-021-09627-2

**Published:** 2021-03-09

**Authors:** Caroline J. Rieser, Richard S. Hoehn, Mazen Zenati, Lauren B. Hall, Eliza Kang, Amer H. Zureikat, Andrew Lee, Melanie Ongchin, Matthew P. Holtzman, James F. Pingpank, David L. Bartlett, M. Haroon A. Choudry

**Affiliations:** 1 Division of Surgical Oncology, Koch Regional Perfusion Center, University of Pittsburgh, UPMC Cancer Pavilion, Pittsburgh, PA USA; 2 AHN Cancer Institute, Allegheny Health Network, Pittsburgh, PA USA

## Abstract

**Background:**

Cytoreductive surgery and hyperthermic intraperitoneal chemoperfusion (CRS HIPEC) can offer significant survival advantage for select patients with colorectal peritoneal metastases (CRPM). Low socioeconomic status (SES) is implicated in disparities in access to care. We analyze the impact of SES on postoperative outcomes and survival at a high-volume tertiary CRS HIPEC center.

**Patients and Methods:**

We conducted a retrospective cohort study examining patients who underwent CRS HIPEC for CRPM from 2000 to 2018. Patients were grouped according to SES. Baseline characteristics, perioperative outcomes, and survival were examined between groups.

**Results:**

A total of 226 patients were analyzed, 107 (47%) low-SES and 119 (53%) high-SES patients. High-SES patients were younger (52 vs. 58 years, *p* = 0.01) and more likely to be White (95.0% vs. 91.6%, *p* = 0.06) and privately insured (83% vs. 57%, *p* < 0.001). They traveled significantly further for treatment and had lower burden of comorbidities and frailty (*p* = 0.01). Low-SES patients more often presented with synchronous peritoneal metastases (48% vs. 35%, *p* = 0.05). Following CRS HIPEC, low-SES patients had longer length of stay and higher burden of postoperative complications, 90-day readmission, and 30-day mortality. Median overall survival following CRS HIPEC was worse for low-SES patients (17.8 vs. 32.4 months, *p* = 0.02). This disparity persisted on multivariate survival analysis (low SES: HR = 1.46, *p* = 0.03).

**Conclusions:**

Despite improving therapies for CRPM, low-SES patients remain at a significant disadvantage. Even patients who overcome barriers to care experience worse short- and long-term outcomes. Improving access and addressing these disparities is crucial to ensure equitable outcomes and improve patient care.

**Supplementary Information:**

The online version contains supplementary material available at. 10.1245/s10434-021-09627-2.

Cytoreductive surgery with hyperthermic intraperitoneal chemoperfusion (CRS HIPEC) is an established locoregional surgical therapy for patients with colorectal peritoneal metastases (CRPM).^1^–^3^ As an invasive and complex procedure, CRS HIPEC carries relatively high morbidity and mortality rates that may impact long-term survival.^4^–^7^ Preoperative evaluation and patient selection are complicated and depend on multiple patient- and tumor-specific characteristics.^7^–^9^ This process is best performed by a multidisciplinary group of specialists with experience managing this disease.

Unfortunately, evolving therapies for complex malignancies such as CRPM have not been available universally to all patients. Patient characteristics such as minority race, rural location, and Medicaid or lack of health insurance have been linked to disparities in treatment and outcomes for complex gastrointestinal malignancies.^10^–^14^ Socioeconomic status (SES), commonly derived from census-level data, has been highlighted as a common factor that may underlie these barriers to treatment.^15^ With regard to colorectal cancer, SES has been correlated with advanced stage at presentation, delays in care, less minimally invasive surgery, and inferior survival.^16^–^18^ However, little is known about the role of SES and the complex management for CRPM.^19^

The goal of the current study is to analyze a large institutional dataset of patients undergoing CRS HIPEC for CRPM and identify any disparities in care and outcomes that may exist as a result of patient SES. This dataset is robust, socioeconomically diverse, and consists of patients from a large geographical region; moreover, it consists of granular pre- and postoperative information along with long-term survival data. Our hypotheses were that low-SES patients would have increased comorbid conditions, decreased access to CRS HIPEC, and inferior short- and long-term outcomes following surgery.

## Patients and Methods

### Study Design and Population

A retrospective cohort study was performed for all patients with CRPM undergoing CRS HIPEC at our institution between 1 January 1 2000 and 31 December 2018 using a prospective database. This study was approved by our institutional review board (IRB 19010278).

The following demographic information was collected for all patients: age (years), sex, race (White, Black, or Asian), primary insurance status (private, Medicare, Medicaid, or none), SES quintile (derived from 2010 US Census data), marital status, employment status (retired, working, unemployed, or disability), distance from treating institution (miles), age-adjusted Charlson–Deyo comorbidity index score (AA-CCI),^20^ modified frailty index (mFI) score,^21^ smoking status (current vs. none), and body mass index (BMI).

The following oncologic variables were examined for all patients: presence of peritoneal metastases at diagnosis of primary tumor (synchronous peritoneal metastases), history of resection of primary tumor, adjuvant chemotherapy receipt following primary tumor resection, pre-CRS HIPEC neoadjuvant chemotherapy, volume of disease quantified by peritoneal cancer index (PCI),^22^ differentiation of tumor (well/moderate vs. poor), signet cell morphology, and perineural invasion (PNI).

The following perioperative factors were collected for all patients: operative time (h), intraoperative blood loss (EBL, mL), completeness of cytoreduction score (CC score 0, 1, 2+),^23^ length of stay (days), in-hospital comprehensive complication index score (CCI),^24^ major complication rates (Clavien–Dindo grade III or higher), readmission within 90 days of discharge to index or other hospital, death within 30 days of CRS HIPEC, and receipt of post-CRS HIPEC chemotherapy.

### Exposure

For this analysis, the primary exposure of interest was low SES, which was determined based on data acquired from the 2010 US Census. ZIP codes were ranked and divided into quintiles based on a SES score as previously described.^25^ Briefly, a composite measure of SES using the US Census American Community Survey 5-year estimates from 2011 was utilized. This measure incorporates measures of wealth, education, and income. Unsurprisingly, the lowest SES quintile had a disproportionately small number of patients. As such, we combined the bottom two quintiles to create a “low-SES” group and compared this with the highest quintile, representing the “high-SES” group.

### Outcome

The primary outcome of interest for this analysis was overall survival (OS) calculated from date of CRS HIPEC to date of death. Secondary outcomes of interest included 90-day readmission and 30-day mortality.

Survival was also considered from date of initial diagnosis of CRPM to death. Progression-free survival (PFS) was also compared between cohorts and defined as time from CRS HIPEC to first clinical or radiographic diagnosis of recurrent or progressive disease following CRS HIPEC. Postprogression survival was also considered from time of first documented progression to death.

### Statistical Methods

Descriptive statistics were used to compare patient characteristics between SES cohorts. Continuous data are reported as median with interquartile range (IQR) and compared using Wilcoxon rank-sum test. Categorical data are reported as frequencies and percentages and compared using chi-square or Fisher exact test as appropriate.

Kaplan–Meier analysis was used to compare survival between SES cohorts and significance determined by log-rank test. To assess the impact of SES on OS following CRS HIPEC, hazard ratio (HR) for low SES was examined using Cox proportional hazard models. Cox proportional hazards models examined impact of race, insurance status, comorbidities, PCI, CC score, tumor differentiation, operative time, blood loss, hospital length of stay, perioperative complications, receipt of post-CRS HIPEC chemotherapy, and repeat CRS HIPEC. Variables with statistical significance *p* < 0.30 on univariate analysis were evaluated in an initial multivariable regression analysis. Variables were sequentially removed via backwards elimination with a prespecified *p *value cutoff of 0.05.

Given the documented differences in outcomes of patients who undergo multiple cytoreductions and potential confounding differences between populations, we conducted a subanalysis of survival between SES cohorts by single and repeat CRS HIPEC status.^26^ We then examined probability of undergoing repeat CRS HIPEC by SES and other baseline patient factors using univariate and multivariate logistic regression. Models were fit as described above.

Missingness of data was minimal (< 1%). An alpha cutoff of 0.05 was used for all significance tests. The data were analyzed using STATA 15 (StataCorp LP, College Station, TX, USA).

## Results

### Patient Demographics and Oncologic Treatment History

A total of 404 patients underwent CRS HIPEC for CRPM during the study period. Overall, 119 patients (29%) were identified as high SES versus 107 (26%) as low SES, resulting in 226 patients being included in the analysis. Median follow-up time was 48.8 months for the whole cohort, with median follow-up of 48.8 months in the high-SES cohort and 50.0 in the low (*p* = 0.61).

Patient demographics and prior oncologic treatment characteristics are presented in Table 1. The low-SES cohort was older (58 vs. 52 years, *p* = 0.01). There was a significant difference in comorbidities, with the low-SES cohort having higher baseline AA-CCI score, mFI score, and BMI (all *p* = 0.01).

**Table 1:** Baseline demographics by socioeconomic status

	High SES *N* = 119	Low SES *N* = 107	*P* value
Age (years)	52 (44–60)	58 (49–66)	0.01
Male	50 (47%)	57 (48%)	0.86
Race			0.06
White	113 (95%)	98 (92%)	
Black	2 (2%)	8 (7%)	
Asian	4 (3%)	1 (1%)	
Smoking	10 (8%)	13 (12%)	0.34
BMI (kg/m^2^)	25.8 (23.2–30.5)	28.2 (24.4–33.2)	0.01
AA-CCI	7 (6–8)	8 (7–9)	0.01
Modified frailty index			
0	89 (75%)	56 (52%)	0.01
1	24 (20%)	33 (31%)	
2+	6 (5%)	18 (17%)	
Employed	77 (65%)	50 (47%)	0.03
Married	81 (68%)	67 (63%)	0.24
Insurance			< 0.001
Private	99 (83%)	61 (57%)	
Medicare	17 (14%)	31 (29%)	
Medicaid	3 (3%)	15 (14%)	
Distance traveled (miles)	310 (23–417)	83 (49–224)	0.01
*Oncologic history*			
Synchronous PM at diagnosis	42 (35%)	51 (48%)	0.05
Prior primary tumor resection	95 (83%)	86 (83%)	0.99
Adjuvant chemotherapy following prior tumor resection	86 (91%)	78 (91%)	0.97
Pre-CRS HIPEC neoadjuvant chemotherapy	108 (92%)	97 (91%)	0.82
Pre-CRS HIPEC weight loss	4 (3%)	8 (7%)	0.15
Pre-CRS HIPEC bowel obstruction	6 (5%)	9 (8%)	0.24

All values depicted as median (IQR) or *n* (%)

*SES* socioeconomic status, *BMI* body mass index, *AA-CCI* age-adjusted Charlson comorbidity index, *PM* peritoneal metastases, *CRS HIPEC* cytoreductive surgery and hyperthermic intraperitoneal chemoperfusion

In considering social determinants of health, the low-SES cohort had lower rates of employment (47% vs. 65%, *p* = 0.03) and higher rates of Medicare and Medicaid insurance (*p* < 0.001). There was a trend towards increasing proportion of minority patients in the low-SES cohort (*p* = 0.06). The high-SES cohort traveled substantially farther to access care (310 vs. 83 miles, *p* = 0.01).

In terms of prior oncologic history, low-SES patients were more likely to have synchronous peritoneal metastases at time of primary diagnosis (48% vs. 35%, *p* = 0.01). Despite this, there were no differences in rates of primary tumor resection, receipt of post-primary resection chemotherapy, or receipt of pre-CRS HIPEC neoadjuvant chemotherapy between cohorts. Importantly, there were no differences in time to CRS HIPEC following diagnosis of CRPM (low SES 6.8 vs. high SES 6.4 months, *p* = 0.89).

### Association of SES with Perioperative Outcomes and Treatment

Perioperative and hospitalization factors by SES status are presented in Table 2. SES cohorts had no difference in preoperative disease burden as assessed by operative PCI score. There were no differences in traditional proxies of operative complexity such as operative time and EBL. Both cohorts achieved complete macroscopic cytoreduction (CC 0) in the majority of cases (low SES: 79% vs. high SES: 80%). From a histological standpoint, there were similar rates of poorly differentiated tumors between cohorts. However, there were higher rates of worrisome features, including signet cell morphology and PNI, in the high-SES cohort.

**Table 2:** Perioperative outcomes by socioeconomic status

	High SES *N* = 119	Low SES *N* = 107	*P* value
*Pathologic findings*			
PCI score	10 (7–19)	11 (8–17)	0.99
Operative time (h)	8.5 (6.8–10.0)	7.7 (6.3–9.4)	0.16
Intraoperative blood loss (mL)	500 (250–750)	500 (300–1000)	0.64
Number of visceral resections	2 (1–3)	3 (2–4)	0.02
Number of anastomoses	1 (0–2)	1 (1–2)	0.1
Ostomy creation	50 (42.0%)	50 (46.7%)	0.48
CC score			0.74
0	95 (80%)	84 (79%)	
1	21 (18%)	22 (20%)	
2	3 (2%)	1 (1%)	
Poorly differentiated	34 (29%)	23 (21%)	0.28
Signet cell morphology	19 (16%)	7 (7%)	0.01
Perineural invasion	34 (29%)	19 (18%)	0.01
*Hospitalization factors*			
Hospital length of stay (days)	11 (8–15)	12 (10–21)	0.01
CCI score	21 (0–31)	23 (9–41)	0.01
Major complications	22 (18%)	23 (22%)	0.73
*Postoperative outcomes*			
90-Day readmission	39 (33%)	52 (51%)	0.01
30-Day mortality	0 (0%)	5 (5%)	0.02
Post-CRS HIPEC adjuvant chemotherapy	64 (60%)	41 (43%)	0.02
Repeat CRS HIPEC	16 (13%)	7 (7%)	0.06

All values depicted as median (IQR) or *n* (%)

*SES* socioeconomic status, *PCI* peritoneal cancer index, *CC score* completeness of cytoreduction score, *CCI* comprehensive complication index

Immediate perioperative outcomes were notably worse in the low-SES cohort, with longer hospitalization (median length of stay 12 vs. 11 days, *p* = 0.01) and more complications (in-hospital CCI score 23 vs. 21, *p* = 0.01). No difference was observed in rates of major perioperative complications (Clavien–Dindo grade III or higher), but 90-day readmission and 30-day mortality rates were significantly higher in the low-SES cohort. All postoperative deaths were in the low-SES group: one patient had a myocardial infarction, two patients had postoperative pneumonia leading to overwhelming sepsis and respiratory failure, and two patients had colonic leaks leading to sepsis and multisystem organ failure. Low-SES patients were also less likely to receive adjuvant chemotherapy following CRS HIPEC (43% vs. 60%, *p* = 0.02). There was a trend towards higher rates of repeat CRS HIPEC among the high-SES cohort, however this did not reach statistical significance (16% vs. 7%, *p* = 0.06).

### Impact of SES on Survival

**Fig. 1:**
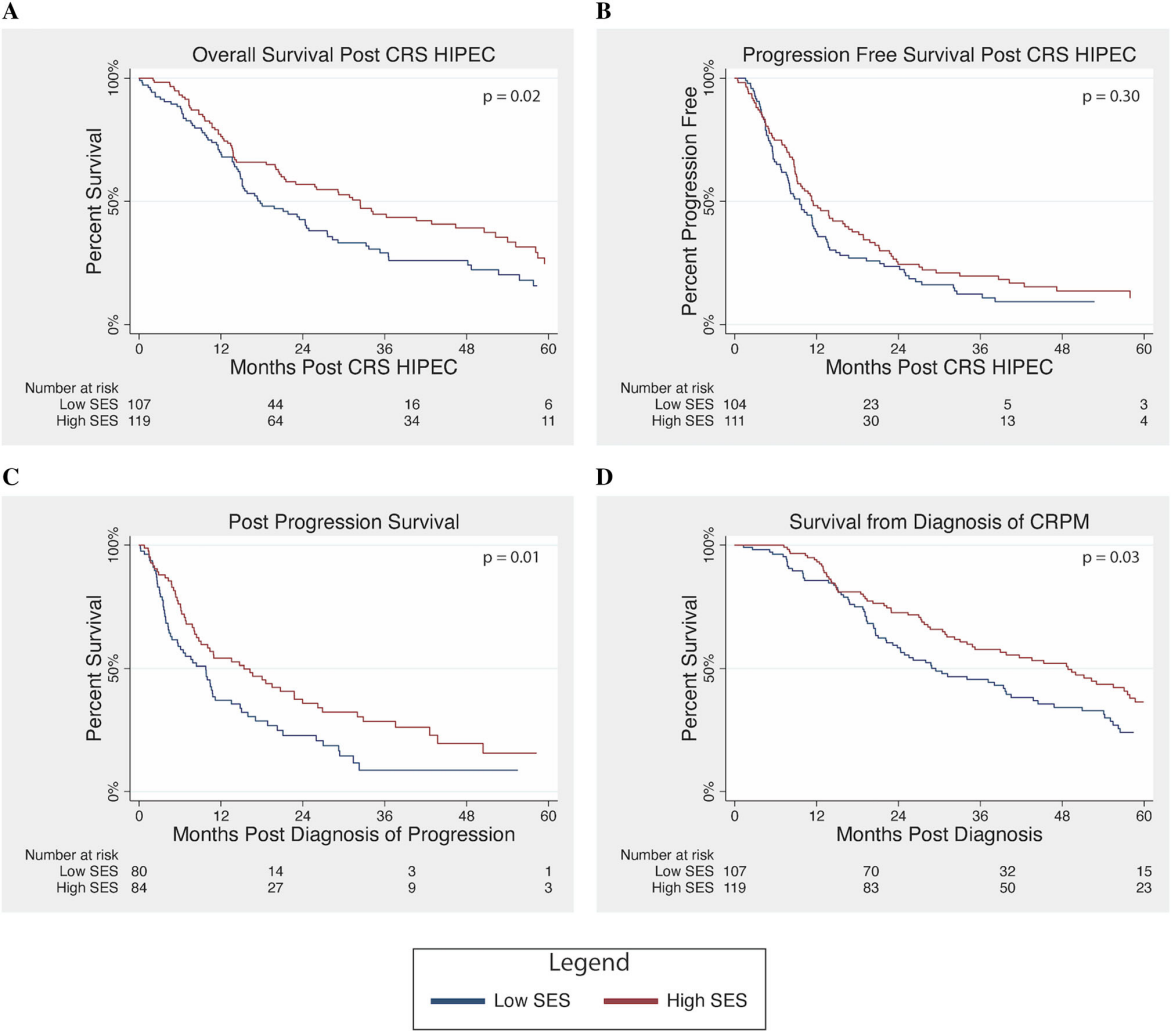
Survival analysis by SES: **a** median overall survival was significantly lower in the low-SES cohort (17.8 versus 32.4 months, *p* = 0.02), **b** there was no difference in progression-free survival following cytoreductive surgery and hyperthermic intraperitoneal chemoperfusion (CRS HIPEC) by SES status (9.5 vs. 11.3 months, *p* = 0.30), **c** post-recurrence overall survival was significantly lower in the low-SES cohort (9.8 vs. 15.4 months, *p* = 0.01), and **d** median overall survival from time of diagnosis of peritoneal metastases was significantly lower in the low-SES cohort (29.5 vs. 48.6 months, *p* = 0.03)

Low-SES patients had significantly worse OS following CRS HIPEC compared with the high-SES cohort, as shown in Fig. 1a (median OS 17.8 vs. 32.4 months, *p* = 0.02). There was no significant difference in PFS following CRS HIPEC (median 9.5 vs. 11.3 months, *p* = 0.30); however, survival postprogression OS was significantly worse for the low-SES cohort (median 9.8 vs. 15.4 months, *p* = 0.01). Survival differences persisted when considering time from diagnosis of CRPM until death, with low-SES patients having significantly worse OS (29.5 vs. 48.6 months, *p* = 0.03).

Univariate analysis revealed that SES, PCI score, CC score, hospital length of stay, major complications, and repeat CRS HIPEC were associated with OS, as presented in Table 3. On multivariate analysis, low SES was an independent predictor of OS (HR 1.46, 95% CI 1.04–2.05, *p* = 0.03). Increasing PCI, CC score, and major complications were also independent predictors of worse OS, while repeat CRS HIPEC and increasing BMI were predictors of improved survival.

**Table 3:** Analysis of factors associated with overall survival following cytoreductive surgery and hyperthermic intraperitoneal chemoperfusion

	Univariate analysis			Multivariate analysis		
	HR	95% CI	*P* value	HR	95% CI	*P* value
*Demographics*						
Low SES	1.43	1.04–1.98	0.03	1.46	1.04–2.05	0.03
Age	1.01	0.99–1.02	0.23			
Male	0.95	0.69–1.31	0.75			
Race						
White	ref		0.60			
Non-white	0.81	0.38–1.74				
Smoking	0.98	0.57–1.68	0.94			
BMI	0.97	0.95–1.00	0.06	0.96	0.93–0.99	0.02
AA-CCI score, per point	1.03	0.94–1.14	0.51			
mFI Score						
0/1	ref					
2+	1.43	0.92–2.22	0.11			
Employed	1.09	0.79–1.50	0.61			
Married	0.98	0.70–1.37	0.90			
Insurance			0.06			
Private	ref					
Medicare	1.45	0.99–2.14				
Medicaid	0.68	0.34–1.34				
Miles traveled	1.00	0.99–1.01	0.13			
*Prior oncologic history*						
PM at diagnosis	1.00	0.72–1.38	0.99			
Pre-CRS HIPEC						
Neoadjuvant						
Chemotherapy	0.91	0.53–1.59	0.75			
*Pathologic findings*						
PCI score, per point	1.11	1.08–1.14	< 0.001	1.09	1.06–1.13	< 0.001
CC score						< 0.001
0	Ref		< 0.001	ref		
1	2.43	1.68–3.51		1.61	1.08–2.39	
2+	6.60	2.37–18.39		5.61	1.99–15.77	
Poorly differentiated	0.83	0.57–1.21	0.33			
Signet morphology	1.11	0.61–2.03	0.74			
PNI	1.27	0.81–2.00	0.30			
*Perioperative factors*						
Hospital length of stay, days	1.04	1.03–1.06	< 0.001			
Major complication	1.98	1.36–2.86	< 0.001	1.49	1.02–2.17	0.04
Post-CRS HIPEC adjuvant chemotherapy	0.82	0.58–1.16	0.27			
Repeat CRS HIPEC	0.49	0.29–0.84	0.01	0.57	0.33–0.99	0.05

*SES* socioeconomic status, *BMI* body mass index, *mFI score* modified frailty index score, *CRS HIPEC* cytoreductive surgery hyperthermic intraperitoneal chemoperfusion, *PM* peritoneal metastases, *PCI* peritoneal cancer index, *CC score* completeness of cytoreduction score, *PNI* perineural invasion

Given the difference in 30-day mortality between groups, OS from CRS HIPEC was also assessed excluding these early mortalities. For this cohort, median OS following CRS HIPEC was 18.0 months (95% CI 15.1–24.9 months) for the low-SES group compared with 32.4 months (95% CI 21.3–46.4 months) for the high-SES group (*p* = 0.05). On multivariate analysis, low SES remained a significant predictor of mortality (HR 1.43, 95% CI 1.01–2.02, *p* = 0.04).

### Repeat CRS HIPEC and SES: Subanalysis of Outcomes and Treatment Receipt

**Fig. 2:**
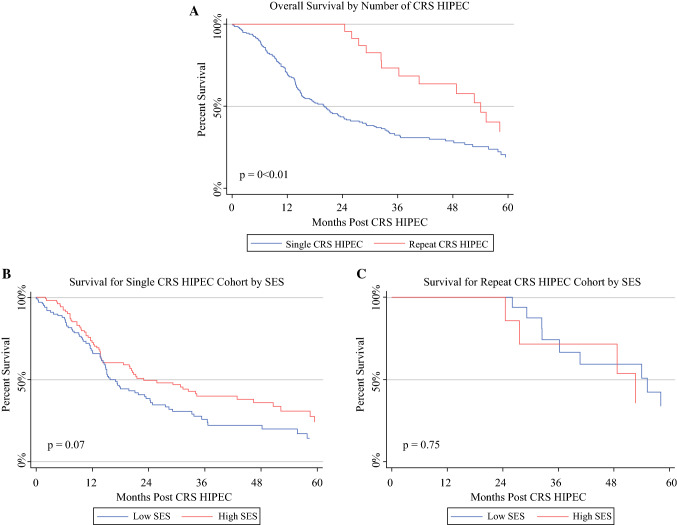
Survival analysis by repeat CRS HIPEC: **a** patients who underwent repeat CRS HIPEC had longer median overall survival compared with those who did not (54.0 vs. 20.0 months, *p* < 0.001), **b** for patients who underwent only one CRS HIPEC procedure, median overall survival was 15.8 months in the low-SES cohort versus 22.9 months in the high-SES cohort (*p* = 0.07), and **c** for patients who underwent repeat CRS HIPEC, there was no difference in median overall survival (52.7 vs. 55.2 months, *p* = 0.75)

A total of 23 patients in our cohort (10.1%) underwent repeat CRS HIPEC procedure during the examined period. Median time to disease progression following first CRS HIPEC for this cohort was 21.2 months (95% CI 13.9–23.7 months). Median time to repeat CRS HIPEC from the date of initial CRS HIPEC was 25.8 months (95% CI 21.8–28.2 months). Repeat CRS HIPEC was associated with improved OS of 54.0 months (95% CI 36.2*–*67.7 months) compared with single CRS HIPEC (20.0 months; 95% CI 15.1–24.4 months, *p* < 0.001). When examined by SES, for the single CRS HIPEC cohort there was a trend towards worse OS for patients with low SES (15.8 months, 95% CI 14.3*–*23.1) compared with high SES (22.9 months, 95% CI 14.2–34.3; *p* = 0.07) (Fig. 2). However, in the repeat CRS HIPEC cohort, there was no difference in survival by SES (52.7 vs. 55.2 months, *p* = 0.75).

Patient-, oncologic-, and treatment-level variables for the repeat CRS HIPEC cohort are presented in Supplementary Table 1. Given documented issues with accessing care faced by patients of low SES, we examined the association of SES and other baseline factors with repeat CRS HIPEC (Table 4). On univariate analysis, BMI and signet cell morphology were significant predictors of repeat CRS HIPEC. Low SES trended towards significance with decreased odds of undergoing repeat CRS HIPEC (OR 0.45, *p* = 0.09).

**Table 4:** Analysis of factors associated with receipt of repeat cytoreductive surgery and hyperthermic intraperitoneal chemoperfusion

	Univariate analysis		
	OR	95% CI	*P* value
*Demographics*			
Low SES	0.45	0.18–1.41	0.09
Age	0.98	0.94–1.01	0.2
Male	0.81	0.34–1.91	0.63
Race			0.65
White	Ref		
Non-white	0.61	0.07–4.89	
BMI	1.07	1.00–1.14	0.04
AA-CCI Score, per point	0.93	0.69–1.24	0.6
Employed	1.89	0.75–4.80	0.18
Married	1.55	0.59–4.12	0.37
Insurance			0.61
Private	Ref		
Medicare	0.53	0.15–1.87	
Medicaid	0.99	0.21–4.64	
Miles traveled	0.99	0.99–1.00	0.32
*Prior oncologic history*			
Peritoneal metastases at diagnosis	1.11	0.47–2.66	0.81
PCI score, per point	0.95	0.88–1.02	0.16
Poorly differentiated	1.34	0.52–3.44	0.55
Signet morphology	3.22	1.14–9.12	0.02
PNI	1.17	0.44–3.14	0.75

*CRS HIPEC* cytoreductive surgery hyperthermic intraperitoneal chemoperfusion, *SES* socioeconomic status, *BMI* body mass index, *AA-CCI* age-adjusted Charlson comorbidity score, *PCI* peritoneal cancer index score, *PNI* perineural invasion

## Discussion

In this study we found that low-SES patients with CRPM experience significant disadvantages with regards to management and outcomes following CRS HIPEC. Low-SES patients had worse baseline comorbidity score, longer postoperative hospital stay, more postoperative complications, and higher readmission rate. Most importantly, low SES was independently associated with inferior long-term survival.

The only published study on patient SES and CRS HIPEC is a 2015 report from Tabrizian et al.^19^ The authors used an institutional dataset to compare 112 patients undergoing CRS HIPEC with patients undergoing colectomy and hepatectomy for colorectal cancer and found that CRS HIPEC patients had higher rates of private insurance and travelled farther for care. On multivariate analysis, CRS-HIPEC was independently associated with younger age, longer distance traveled, and type of insurance compared with the other two procedures. This study demonstrated that increasing complexity of colorectal disease was associated with decreased access for low-SES and non-White patients. However, this study did not directly compare high- versus low-SES patients and there was no investigation of patient characteristics, surgical outcomes, or survival.

This is the first study to identify disparities in outcomes following CRS HIPEC when comparing low- versus high-SES patient cohorts. The disparities identified are nuanced and warrant further examination. Low-SES patients presented with higher BMI and significantly worse Charlson–Deyo comorbidity and mFI scores. They also presented more often with synchronous peritoneal metastases, suggesting delayed presentation and potential decreased screening utilization. However, other cancer characteristics and oncologic treatments were similar between groups. Peritoneal cancer index, CC score, and operative complexity were similar between SES groups. Pathologic tumor assessment showed similar tumor grades and actually found high-SES patients to have more frequent signet ring cells and PNI. These findings are important because “biologic differences” are frequently suggested as an explanation for racial, ethnic, and other socioeconomic disparities in cancer outcomes. Higher rates of worrisome features in the high-SES cohort could indicate referral biases for CRS HIPEC. Low-SES patients with worrisome features may be more likely to be referred to systemic chemotherapy or less able to travel and obtain a second opinion.

Our analysis suggests that inferior long-term survival for low-SES patients following CRS HIPEC is not explained by known tumor prognostic factors. On multivariate analysis, inferior survival was associated with low SES, PCI score, CC score, and major postoperative complications. It is interesting to note that PFS was similar between groups but OS from diagnosis and from surgery were still inferior for low-SES patients. Differences in survival appear to be driven by two main phases. First, differences in immediate mortality are likely driven by complications. While overall complication rates are similar, older age and worse baseline health as suggested by AA-CCI and mFI may place low-SES patients at higher risk for failure to rescue following major postoperative complications. Failure-to-rescue rates, defined as rate of mortality following at least one major complications, have been shown to be higher for low-SES patients following resection.^27^ Similarly, in this analysis we find failure-to-rescue rates of 0% for the high-SES cohort compared with 17.4% for low-SES patients (*p* = 0.05). After the immediate postoperative period, given similar time to progression in both cohorts, survival differences appear to result from differences in long-term disease management. Decreased receipt of adjuvant chemotherapy following CRS HIPEC and lower rates of repeat CRS HIPEC at disease recurrence may account for some of the observed survival differences. Importantly, on multivariate analysis, SES remained a predictor of mortality even after accounting for receipt of adjuvant chemotherapy and repeat CRS HIPEC. These findings hint at unmeasured differences that may occur in the longitudinal management of these patients. Future studies may help elucidate these differences and identify areas for improvement.

In our exploratory analysis of predictors of repeat CRS HIPEC, BMI and signet ring cell morphology were significant predictors on univariate analysis while SES trended towards significance. The unexpected finding of signet cell morphology having a positive association with repeat CRS HIPEC is likely due to selection bias. While the sample size is small, there is a suggestion of stricter criteria for operating on the signet ring patients. Patients were younger (median age 47 vs. 56 years, *p* = 0.07), traveled further for care (190 vs. 126 miles, *p* = 0.20), had lower rates of frailty (7.7% vs. 15.5%, *p* = 0.23), and had more high-SES patients (73.9% vs. 50.0%, *p* = 0.02). This unexpected finding requires further validation in larger cohort studies, but points to the complex interplay of patient and oncologic factors that drive treatment receipt.

There are of course limitations to this study. While the dataset is large and prospectively collected, this study is a retrospective analysis of data from a single institution and thus cannot assert more than a correlation between SES and patient outcomes. We lack cancer-specific survival data, and given differences in age and comorbidities between SES cohorts, it is possible that some of the increased mortality burden is due to underlying health conditions. Another potential limitation is the use of census-level data to estimate SES rather than directly reported patient characteristics. However, these methods have been found in numerous studies to reliably estimate patient SES along with important health-related outcomes.^28^

In conclusion, this study highlights important disparities that exist for low-SES patients with complex diseases such as CRPM. Low-SES patients are limited in their ability to travel for specialized care and suffer worse surgical outcomes, potentially due to increased comorbidities. Low-SES patients also have inferior long-term survival that is not necessarily explained by cancer biology and may be a further consequence of limited resources. As the multidisciplinary management of complex and metastatic malignancies continues to evolve, it is imperative that comprehensive treatment strategies be made more available to all patients.

## Supplementary Information

Below is the link to the electronic supplementary material.

Supplementary file1 (DOCX 24 kb)
