# Supplementary material for: Impact of Socioeconomic Status on Presentation and Outcomes in Colorectal Peritoneal Metastases Following Cytoreduction and Chemoperfusion: Persistent Inequalities in Outcomes at a High-Volume Center
Source: Ann Surg Oncol. 2021 Mar 9;28(7):3522–31. doi: 10.1245/s10434-021-09627-2 (PMC8184539; doi:10.1245/s10434-021-09627-2)
Supplement: Supplementary file 1 — Supplementary file1 (DOCX 24 kb) [file 10434_2021_9627_MOESM1_ESM.docx]

| **Supplemental Table 1: Patient, Oncologic, and Treatment Factors by Repeat CRS HIPEC Status** | | | |
| --- | --- | --- | --- |
|  | **No Repeat CRS HIPEC**  **(n=203)** | **Repeat CRS HIPEC (n=23)** | **P Value** |
| Age | 55.9 (46.5-63.1) | 48.1 (44.8-61.4) | 0.15 |
| Male | 108 (53.2%) | 11 (47.8%) | 0.63 |
| Non-White Race | 14 (6.9%) | 1 (4.4%) | 0.001 |
| BMI | 26.3 (23.5-30.7) | 31.0 (24.9-35.5) | 0.03 |
| AA-CCI | 7 (7-9) | 7 (6-9) | 0.33 |
| Modified Frailty Index |  |  | 0.022 |
| 0 | 170 (83.7%) | 23 (100%) |  |
| 1 | 33 (16.3%) |  |  |
| High SES | 103 (50.7%) | 16 (69.6%) | 0.07 |
| Employed | 111 (54.7%) | 16 (69.6%) | 0.13 |
| Married | 131 (64.5%) | 17 (73.9%) | 0.26 |
| Insurance |  |  | 0.29 |
| Private | 142 (70.0%) | 18 (78.3%) |  |
| Medicare | 45 (22.2%) | 3 (13.0%) |  |
| Medicaid | 16 (7.9%) | 2 (8.7%) |  |
| Distance traveled, miles | 131 (43-370) | 143 (55-387) | 0.98 |
| **Oncologic History and Pathologic Findings** | |  |  |
| Synchronous PM at diagnosis | 83 (40.9%) | 10 (43.4% | 0.49 |
| PCI Score | 12 (9-14) | 12 (8-12) | 0.27 |
| CC Score |  |  | 0.86 |
| 0 | 161 (79.3%) | 18 (78.3%) |  |
| 1 | 38 (18.7%) | 5 (21.7%) |  |
| 2 | 4 (2.0%) |  |  |
| Poorly differentiated | 50 (24.6%) | 7 (30.4%) | 0.35 |
| Signet cell morphology | 20 (9.9%) | 6 (26.1%) | 0.03 |
| Perineural invasion | 47 (23.2%) | 6 (26.1%) | 0.46 |
| **Postoperative Outcomes** | | | |
| Major Complications | 42 (20.7%) | 3 (13.0%) | 0.28 |
| Post-CRS HIPEC Adjuvant Chemotherapy | 86 (42.4%) | 19 (82.6%) | 0.001 |

SES, Socioeconomic Status; BMI, Body Mass Index; mFI score, modified Frailty Index Score; CRS HIPEC, Cytoreductive Surgery Hyperthermic Intraperitoneal Chemoperfusion; PM, Peritoneal Metastases; PCI, Peritoneal Cancer Index; CC Score, Completeness of Cytoreduction Score; PNI, perineural invasion
